# Inhibitory Effects of Total Triterpenoid Saponins Isolated from the Seeds of the Tea Plant *(Camellia sinensis)* on Human Ovarian Cancer Cells

**DOI:** 10.3390/molecules22101649

**Published:** 2017-09-30

**Authors:** Ling-Yan Jia, Xue-Jin Wu, Ying Gao, Gary O. Rankin, Alexa Pigliacampi, Heather Bucur, Bo Li, You-Ying Tu, Yi Charlie Chen

**Affiliations:** 1 Department of Tea Science, Zhejiang University, Hangzhou 310058, China; 11416013@zju.edu.cn (L.-Y.J.); wuxuejin@zju.edu.cn (X.-J.W.); drlib@zju.edu.cn (B.L.); 2 College of Science, Technology and Mathematics, Alderson Broaddus University, Philippi, WV 26416, USA; pigliacampiam@battlers.ab.edu (A.P.); bucurhm@battlers.ab.edu (H.B.); 3 Tea Research Institute, Chinese Academy of Agricultural Sciences, Hangzhou 310008, China; yinggao@tricaas.com; 4 Department of Pharmacology, Physiology and Toxicology, Joan C. Edwards School of Medicine, Marshall University, Huntington, WV 25755, USA; rankin@marshall.edu

**Keywords:** triterpenoid saponins, ovarian cancer, apoptosis, angiogenesis, *Camellia sinensis*

## Abstract

Ovarian cancer is regarded as one of the most severe malignancies for women in the world. Death rates have remained steady over the past five decades, due to the undeniable inefficiency of the current treatment in preventing its recurrence and death. The development of new effective alternative agents for ovarian cancer treatment is becoming increasingly critical. Tea saponins (TS) are triterpenoidsaponins composed of sapogenins, glycosides, and organic acids, which possess a variety of pharmacological activities, and have shown promise in the anti-cancer field. Through cell CellTiter 96^®^ Aqueous One Solution Cell Proliferation assay (MTS) assay, colony formation, Hoechst 33342 staining assay, caspase-3/7 activities, flow cytometry for apoptosis analysis, and Western blot, we observed that TS isolated from the seeds of tea plants, *Camellia sinensis*, exhibited strong anti-proliferation inhibitory effects on OVCAR-3 and A2780/CP70 ovarian cancer cell lines. Our results indicate that TS may selectivity inhibit human ovarian cancer cells by mediating apoptosis through the extrinsic pathway, and initiating anti-angiogenesis via decreased VEGF protein levels in a HIF-1α-dependent pathway. Our data suggests that, in the future, TS could be incorporated into a potential therapeutic agent against human ovarian cancer.

## 1. Introduction

Ovarian cancer is the fifth leading cause of cancer-related death from gynecological malignancy [1]. Epithelial ovarian carcinoma is the major form of the disease and accounts for approximately 90% of all ovarian tumors [2]. In 2016, approximately 22,280 new cases of epithelial ovarian cancer were diagnosed and 14,240 deaths occurred in the United States [3]. Prevention of ovarian cancer and early diagnosis continues to be challenging due to the lack of ovarian cancer symptoms and effective screening procedures. Nearly 75% of ovarian cancer patients have advanced stages of the disease and 70% of patients diagnosed experience recurrence within five years with survival rates of only 17–36% [4,5]. Currently, the combination of surgical cytoreduction surgery with platinum-based chemotherapy is the main clinical application for solid tumors [6]. Cisplatin is the most effective and widely used chemotherapeutic agent employed for treatment against head, neck, ovarian, testicular, lung, and others types of cancer [7,8]. However, in most cases, chemoresistance is regarded to be responsible for treatment failure [9]. Unacceptable rates of normal cell toxicity and various side-effects result from chemotherapies, such as nephrotoxicity, neurotoxicity, hemolytic anemia, myelotoxicity, nausea, and vomiting. Thus, the discovery of novel and more effective agents is urgent for patients with ovarian cancer.

Cancer is a dynamic, variable, and multistep process that marks the conversion of normal cells to malignant cells. Alterations in the apoptotic process have been demonstrated as having a strong relationship to cancer [10]. Apoptosis, an ordered and orchestrated cellular death process, usually occurs under pathological and physiological conditions [11]. Recent studies show that the dysregulation of apoptosis is found in a wide spectrum of human diseases, including cancer. Numerous studies have indicated that the failure of molecular mechanisms under apoptosis in chemoresistant and/or advanced ovarian cancer cells include the down-regulation of pro-apoptotic proteins Puma, Bax, and Bad, and the up-regulation of the anti-apoptotic protein Bcl-2 family including, Bcl-2 and Bcl-xL. One of the potential strategies to overcoming drug resistance in ovarian cancer cells is to increase the susceptibility of cells to apoptosis [12,13].

Angiogenesis, the formation of new, irregular blood vessels apart from a preexisting vascular network [14], plays a key role in the growth and progression of carcinomas [15]. Carcinoma vasculatures usually contain poor blood flow and high vascular permeability, which leads to decreased uptake of cytotoxic drugs and increased potential for metastasis [16]. Studies have demonstrated that multiple molecules and growth factors, such as hypoxia-inducible factor 1 (HIF-1) and vascular endothelial growth factor (VEGF), control angiogenesis [17]. The HIF-1 protein belongs to a basic helix-loop-helix Per Ant Sim (PAS) protein family [18], which consists of two subunits, a hypoxiaregulated α subunit (HIF-1α) and a non-hypoxia-regulated β subunit (HIF-1β) [19]. VEGF is not only the most crucial angiogenic factor in modulating multiple steps, but is also the target of anticancer chemotherapy agents. HIF-1 regulates the activation of VEGF [20]. In over 70% of human cancers and metastases, overexpression of HIF-1α has been verified [21]. For this reason, it is important that anticancer chemotherapy agents target HIF-1α and VEGF proteins.

Saponins are plant secondary metabolites that exist throughout the plant kingdom and have diverse biologic activities [22]. Tea saponins are oleanane-type triterpenoid saponins isolated from various parts of tea plants (*Camellia sinensis*), including seeds [23,24,25], leaves [26], flowers [27], and roots [28]. In the past decades, multiple reports have been published on the separation and structure elucidation of saponin monomers, such as teaseedsaponin A–L, triterpene saponins S_1_–S_4_, 21-*O*-Angeloyltheasapogenol E_3_, theasaponins A_1_–A_9_, theasaponins B_1_, B_5_, theasaponins C_1_, theasaponins E_1_–E_13_, theasaponins F_1_–F_3_, theasaponins G_1_ and G_2_, and theasaponins H_1_ from the seeds of *Camellia sinensis* (L.) O. Kuntze [24,29,30,31,32]. TS has been demonstrated as being an excellent bio-surfactant with numerous pharmacological activities. TS has been documented as having gastroprotective [33], anti-fungal [34], anti-viral [25], anti-oxidative [24], anti-obesity [35], anti-inflammatory [24,28], anti-hyperglycemic [27], immunomodulatory [36], and anti-tumor [35,37,38] tendencies. However, unlike other phenolic compounds, EGCG [39,40,41] and TFs [37,42] separated from tea plants, TS has not been reported as having pharmacological influences on human ovarian cancer. In the present study, the cellular and molecular inhibitory effects of TS on both platinum-resistant ovarian cancer cell lines OVCAR-3 and A2780/CP70 in vitro were explored.

## 2. Results

### 2.1. TS Inhibits Cell Growth and Colony Formation In Vitro

**Figure 1:**
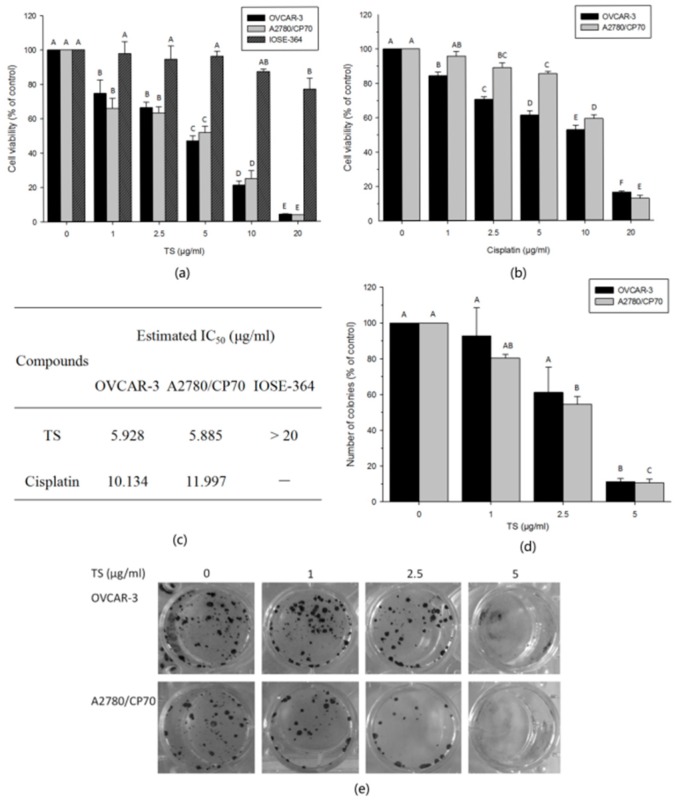
Effects of TS on cell growth and colony formation in vitro in OVCAR-3 and A2780/CP70 human ovarian cancer cells and the cytotoxicity of TS on IOSE-364 normal ovarian cells. (**a**) TS inhibits cell viability of OVCAR-3, A2780/CP70 and IOSE-364 cells after 24 h. Cell viability was determined via MTS assay; (**b**) Cisplatin inhibits cell growth of OVCAR-3 and A2780/CP70 cells after 24 h as a control compared with TS’s inhibitory activity; (**c**) The estimated half-maximal inhibitory concentration (IC_50_) of TS and cisplatin against ovarian cancer cells and/or normal ovarian cells; (**d**) Colony formation activity of OVCAR-3 and A2780/CP70 cells was inhibited by TS at 24 h; (**e**) TS exhibited extensive colony formation inhibitory effects in OVCAR-3 and A2780/CP70 cells at 24 h. The capital letters (A, B, etc.) mean extremely significant differences among different treatments (*p* < 0.01).

To investigate the anti-proliferation effect of TS, MTS assay was performed post TS treatment on both ovarian cancer and normal ovarian epithelial cells. Meanwhile, we chose cisplatin as the medicinal control. TS treatments were compared with cisplatin on OVCAR-3 and A2780/CP70. The cytotoxic activity of TS on IOSE-364 was compared with that on ovarian cancer cells. The results showed that, for both cancer cell lines, TS treatment significantly decreased their cell viability in a dose-dependent manner. However, TS showed less cytotoxic vitality on IOSE-364 cells (Figure 1a, *p* < 0.01). MTS data revealed that the percentage of viable OVCAR-3 cells ranged from 74.6% to 4.1%; meanwhile, A2780/CP70 cells ranged from 66.0% to 3.7%, and IOSE-364 cells ranged from 97.7% to 76.8% upon exposure to TS for 24 h at concentrations ranged from 1 to 20 µg/mL (Figure 1a, *p* < 0.01). The IC_50_ values of TS treated OVCAR-3, A2780/CP70 and IOSE-364 cells were estimated to be 5.9 µg/mL, 5.9 µg/mL and over 20 µg/mL, respectively (Figure 1c). While the percent of viable OVCAR-3 cells treated with cisplatin varied from 84.4% to 16.4%, and A2780/CP70-viable cells from 95.8% to 12.9% (Figure 1b, *p* < 0.01). Shown in Figure 1c, the IC_50_ values of cells treated with cisplatin were 10.1 µg/mL for OVCAR-3 and 11.9 µg/mL for A2780/CP70. For both human ovarian cancer cell lines, the IC_50_ values of TS treatments were approximately half the IC_50_ values of cisplatin treatments. Our results revealed that TS exerts a more potent inhibitory effect on cell proliferation than cisplatin for both OVCAR-3 and A2780/CP70 cells. Compared to ovarian cancer cells, TS showed a lower cytotoxic effect against normal ovarian epithelial cells. 

The colony forming ability of each cell line was determined to explore if TS had the ability to inhibit cell colony formation in vitro. The results from Figure 1d,e showed that both OVCAR-3 and A2780/CP70 cells treated with TS at various concentration rates from 1 to 5 µg/mL, formed fewer colonies compared to the control group of cells in a dose-dependent manner, especially at 5 µg/mL (Figure 1d, *p* < 0.01). This finding was consistent with the MTS assay results. Under these conditions, the potent inhibitory activity of TS on cell growth and colony formation on both OVCAR-3 and A2780/CP70 cancer cells was demonstrated.

### 2.2. TS Induces Apoptosis in Both OVCAR-3 and A2780/CP70 Cells

**Figure 2:**
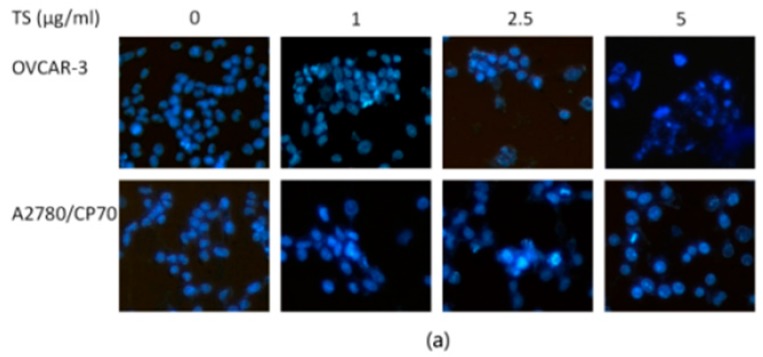

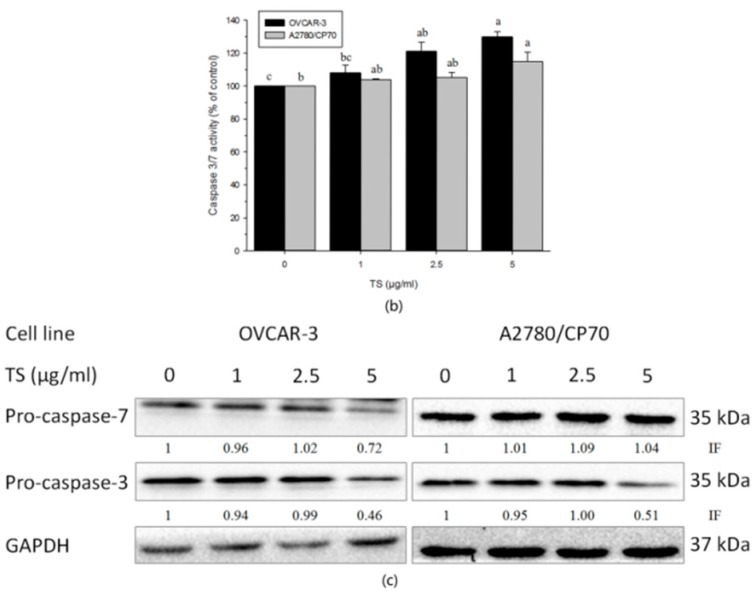
Apoptosis-inducing effects of TS on OVCAR-3 and A2780/CP70 cells. (**a**) Hoechst 33342 staining of OVCAR-3 and A2780/CP70 cells after treatment with 0 to 5 µg/mL TS for 24 h via fluorescent microscopy (200×). Intact nuclei represents viable cells and highly condensed or fragmented nuclei represent apoptotic cells; (**b**) Caspase-3/7 activity levels after TS treatment (0 to 5 µg/mL) for 24 h. The caspase-3/7 activity of control group was arbitrarily expressed as 100%; (**c**) Protein expression levels of pro-caspase-3 and -7 determined by Western blotting assay. Data represents mean ± SD of three independent experiments. Lower case letters (a, b, etc.) mean significant differences among different treatments (*p* < 0.01). “IF” means impact factor and represents the Western blotting signals were quantified and normalized to the loading control GAPDH.

**Figure 3:**
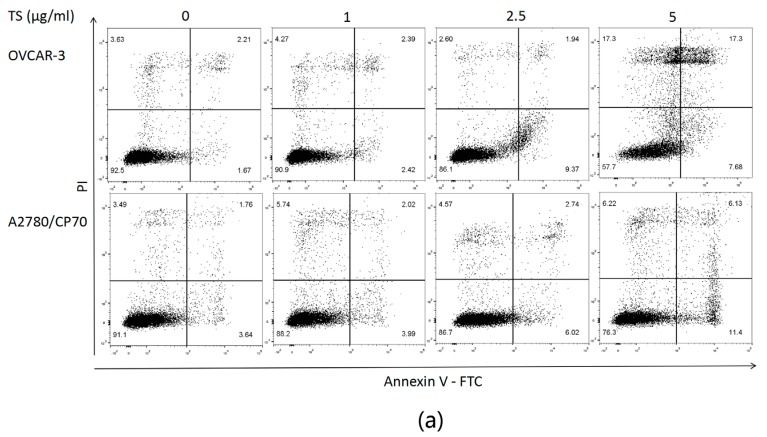

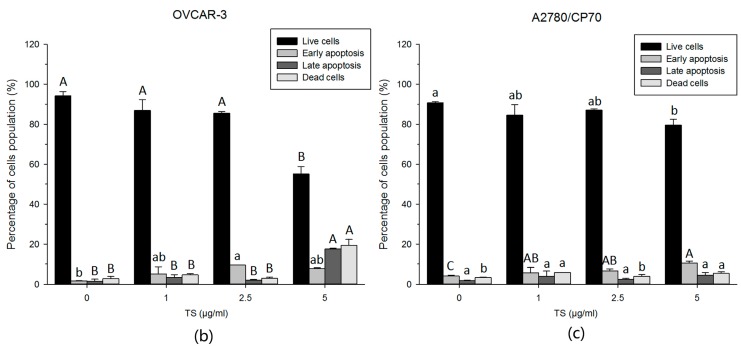
TS induces apoptosis in OVCAR-3 and A2780/CP70 cells. (**a**) Flow cytometry of TS treated OVCAR-3 and A2780/CP70 cells using a double staining method with FITC-conjugated Annexin V and PI. The upper left and lower left quadrant indicate the percentage of dead and live cells, respectively. The upper right and lower right quadrants indicate the percentage of late and early apoptotic cells, respectively; (**b**) Bar graphs showing the percentage of cell population of live cells, early apoptosis cells, late apoptosis cells and dead cells with treatment 0 to 5 µg/mL TS in OVCAR-3; (**c**) A2780/CP70. The capital letters (A, B, etc.) denote extremely significant differences among different treatments (*p* < 0.01); and lower case letters (a, b, etc.) mean significant differences among different treatments (*p* < 0.05).

Nuclear morphology changes of OVCAR-3 and A2780/CP70 cells treated with TS (0 to 5 µg/mL) for 24 h were examined to investigate whether TS inhibited cell viability by inducing apoptosis in platinum-resistant ovarian cancer cells. Cells were treated using Hoechst 33342 staining assay and analyzed under a fluorescent microscope after exposure to TS for 24 h. The results showed that both ovarian cancer cell lines treated with TS exhibited more apoptotic cells with condensed or fragmented nuclei than untreated groups. Nuclei stained were less bright and intact (Figure 2a). The number of cells in both cell lines also decreased after TS treatment (Figure 2a). This result was consistent with the result of the MTS assay (Figure 1a) and colony formation assay (Figure 1d,e). To confirm that TS induced apoptosis, caspase-3/7 enzymatic activities were evaluated by a Caspase-Glo 3/7 Assay Kit, an Annexin V FITC, and PI double stain, followed by flow cytometry analysis. The protein levels were then detected via western blot analysis for both ovarian cancer cell lines. Figure 2b revealed that the treatment with TS increased the caspase-3/7 enzymatic activity to 1.29 and 1.15 times that of the controls for OVCAR-3 and A2780/CP70 cells, respectively (Figure 2b, *p* < 0.05). Meanwhile, the treatment of TS decreased the protein expression of pro-caspase-3 in both cell lines and only decreased pro-caspase-7 in OVCAR-3 cells (Figure 2c). As shown in Figure 3, TS decreased the number of living cells and increased the number of early apoptotic cells in both cell lines. Figure 3b,c showed that TS at 5 µg/mL, significantly increased the total percent of apoptotic cells (upper right quadrant + low right quadrant) from 3.13% to 25.44% and 5.97% to 15.03%, with concentrations ranging from 0 to 5 µg/mL in OVCAR-3 and A2780/CP70 cells, respectively. The TS-induced growth inhibitory effect was demonstrated and had the potential to induce apoptosis at varying strengths for both OVCAR-3 and A2780/CP70 cells.

### 2.3. Effect of TS on the Intrinsic Apoptotic Pathway 

**Figure 4:**
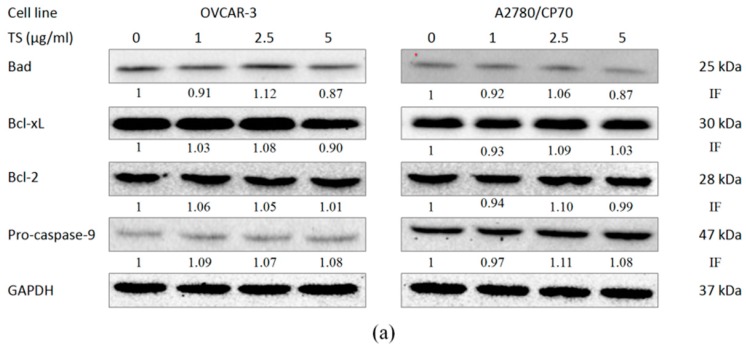

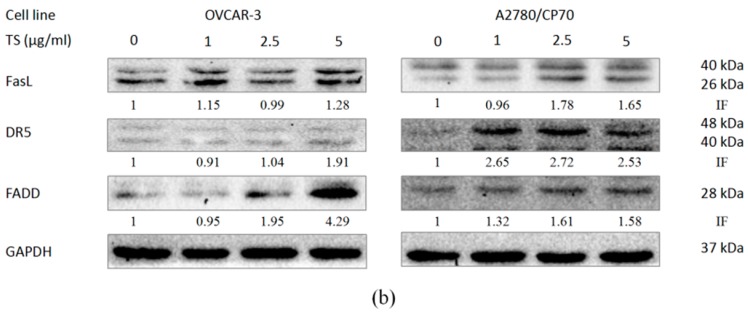
Effects of TS on the intrinsic apoptotic pathway and extrinsic apoptotic pathway in OVCAR-3 and A2780/CP70 cells. (**a**) The intrinsic apoptotic pathway related protein expression levels of Bad, Bcl-2, Bcl-xL, and pro-caspase-9 with 0 to 5 µg/mL TS treatment for 24 h by Western blotting; (**b**) The extrinsic apoptotic pathway-related protein expression levels of FasL, DR5, and FADD after 0 to 5 µg/mL TS treatment for 24 h by Western blotting. “IF” means impact factor and represents the Western blotting signals were quantified and normalized to the loading control GAPDH.

The initiation of apoptosis in cancer cells can be separated into two pathways: the intrinsic (mitochondrial) pathway and the extrinsic (death receptor) pathway [43]. To determine if the intrinsic pathway is involved in TS-induced apoptosis, the protein expression of pro-apoptotic Bcl-2 family proteins (Bad), anti-apoptotic Bcl-2 family proteins (Bcl-2 and Bcl-xL), and pro-caspase-9 in OVCAR-3 and A2780/CP70 cell lines were examined by Western blotting analysis. Figure 4a showed that expression of pro-apoptotic proteins Bad, anti-apoptotic proteins Bcl-2, Bcl-xL and pro-caspase-9 was not significantly affected by TS treatment. These results indicate that TS might induce apoptosis in OVCAR-3 and A2780/CP70 cells not via the intrinsic apoptotic pathway, but through the Bcl-2 protein family.

### 2.4. Effect of TS on the Extrinsic Apoptotic Pathway 

To investigate if the extrinsic pathway is involved in TS-induced apoptosis the expression of FasL, DR5 and FADD in OVCAR-3 and A2780/CP70 cell lines was examined by Western bolt analysis. The results revealed that TS increased the expression of matured DR5 and FADD in both cell lines. The FasL protein levels was increased only in A2780/CP70 cells (Figure 4b). These results suggest that TS might induce apoptosis in OVCAR-3 and A2780/CP70 cells through an extrinsic-independent pathway associated with FADD and DR5 proteins with different strengths of inhibitory effects between the cell lines.

### 2.5. TS Reduces Tumor-Induced Angiogenesis In Vitro by Downregulating VEGF and HIF-1α

**Figure 5:**
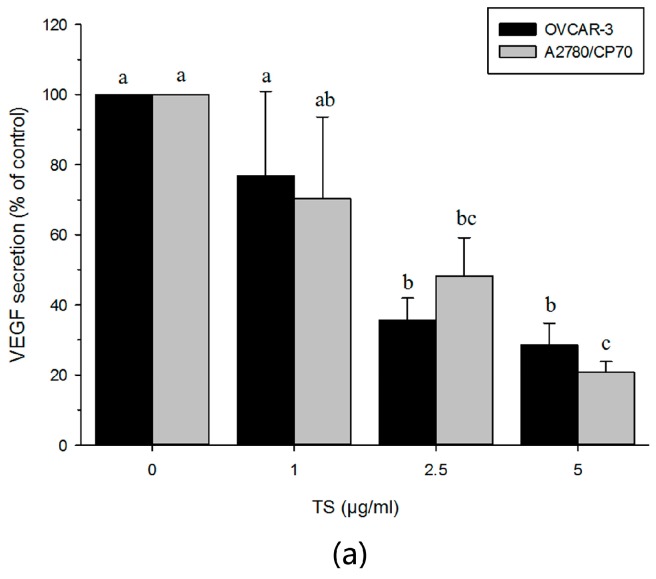

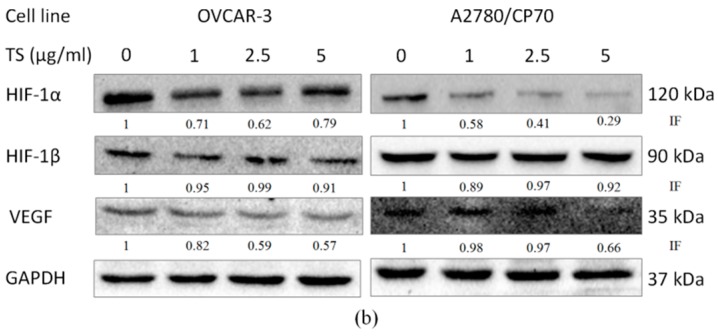
Effects of TS on anti-angiogenesis via decreased VEGF secretion and protein expression levels of VEGF and HIF-1α. (**a**) The protein secretion of VEGF in TS-treated OVCAR-3 and A2780/CP70 cell culture supernatant was detected via VEGF ELISA assay. Lower case letters (a, b, etc.) mean a significant differences among different treatments (*p* < 0.05); (**b**) The protein expression levels of HIF-1α, HIF-1β and hVEGF with 0 to 5 µg/mL treatment TS for 24 h analyzed by Western blotting. “IF” means impact factor and represents the Western blotting signals were quantified and normalized to the loading control GAPDH.

A VEGF ELISA assay confirmed the reduction of VEGF in a culture supernatant form through TS treatment (Figure 5a, *p* < 0.05). Our results showed that TS treatments diminished the secretion of VEGF. VEGF secretion was decreased from 76.8% to 28.6% in OVCAR-3 cells and from 70.3% to 20.7% in A2780/CP70 cells when treated with 1 to 5 µg/mL of TS. Western blotting analysis revealed that TS decreased the expression of hVEGF and HIF1α, but had no influence on the expression of HIF-1β (Figure 5b). These results suggest that TS reduces VEGF expression through a HIF1α-dependent pathway rather than through an HIF-1β pathway.

## 3. Discussion

Ovarian cancer is a lethal gynecologic disease of the female reproductive tract [2] with most patients still suffering from relapse, chemoresistance, and adverse side effects [44]. Cytoreductive surgery followed by chemotherapy, the current treatment option, generally remains ineffective for late cancer stages with a five-year survival rate of less than 40% [4]. No remarkable changes in death rates in the past five decades further presents the urgent necessity for new chemotherapy strategies. Recently, the understanding of tumor progression has significantly improved and has been used to develop molecular biology and targeting agents. Research efforts strive to identify alternative agents that target tumor microenvironments to influence angiogenesis, apoptosis, invasion, and metastasis processes [44].

Previous studies have found that natural compounds may be used as effective chemotherapeutic agents because they have few side effects [45]. TS has been studied for its pharmacological activities since 1931. For instance, Theasaponin E_1_ has been theorized as a possible cancer treatment option because of its anti-angiogenesis mechanisms [35]. Chakasaponins I and II and floratheasaponin A showed anti-proliferative activities through apoptotic-induced cell death by activation of caspase-3/7 in HSC-2 cells [38]. Little research has been published on tea saponins beneficial biological activities on ovarian carcinoma. However, many investigations have been undertaken to better understand the influence of saponins isolated from other plants on ovarian cancer including 20(S)-protopanaxadiol saponins [46], paris saponin VII [47], paris saponin II [48,49,50], and ginsenoside 20(S)-Rg3 [51,52,53]. These findings provoked interest in determining the inhibitory activities of TS on the human ovarian cancer cells. This research tested the cytotoxicity of TS on ovarian tumor cells and found that TS exhibited a more potent inhibitory effect on OVCAR-3 and A2780/CP70 ovarian cancer cell lines than cisplatin, and exhibited lower cytotoxicity towards the normal cell line than ovarian cancer cells. Capase-3/7 activities, flow cytometry for apoptosis assay, and Western blotting analysis showed that the anti-proliferation activities of TS on cisplatin-resistant ovarian cancer cell lines might be attributed to inducing apoptosis and anti-angiogenesis.

Apoptosis, programmed cell death, plays a crucial role in human development, immunity, and tissue homeostasis [54]. Many studies have found that a decreased susceptibility of ovarian cancer cells to apoptosis was strongly associated with drug resistance. Therefore, increasing the susceptibility of cancer cells to apoptosis is one of the potential strategies used to overcome drug resistance in cancer treatment [55]. There are two primary pathways that lead to apoptosis, including the intrinsic pathway and the extrinsic pathway [54]. The intrinsic apoptotic pathway is regulated by the Bcl-2 protein family. Proteins in this family exhibit either pro-apoptotic or anti-apoptotic activities that control the permeability of the mitochondrial membrane and the release of cytochrome c into the cytoplasm [43]. The balance between pro-apoptotic and anti-apoptotic proteins determines if cells will undergo apoptosis. Cytochrome c forms an apoptosome complex associated with pro-caspase-9, dATP, and APAF-1 to initiate the activation of pro-caspase-9 [54,55]. In the extrinsic pathway, tumor necrosis factor relates apoptosis-promoting members such as Apo2L/TRAIL and FasL, which engage their respective death receptors DR5. These death receptors homotypically bind to the adaptor protein FADD. Activation of either the intrinsic or extrinsic pathway results in the activation of caspases, which cleaves downstream pro-caspases, such as pro-caspase-3, 6, and 7 to finally trigger cell death [54]. The present research found an up-regulated expression of proteins DR5 and FADD, down-regulated protein pro-caspase-3, and had no influence on Bcl-2, Bcl-xL, Bad, and pro-caspase-9 in both OVCAR-3 and A2780/CP70 cells. Additionally, caspase-3/7 activities significantly increased with 5 µg/mL TS treatment. These results indicate that the anti-proliferation activity of TS might be due to the induction of apoptosis through the extrinsic pathway, but not through the intrinsic pathway. It is proposed that TS might be a promising chemotherapeutic agent for platinum-resistant ovarian cancer, as TS induced apoptosis in both ovarian cancer cell lines through the reduction of cell viability and the percentage of apoptotic cells. However, although our results indicated that TS induced apoptosis in both OVCAR-3 and A2780/CP70 cells, a significantly higher reduction of cell viability (53.05% and 48.25%, respectively, at 5 µg/mL TS) with respect to the percentage of apoptotic cells, including early and late apoptosis cells (25.44% and 15.03%, respectively, at 5 µg/mL TS) demonstrated other mechanisms likely influence the cell proliferation-inhibitory activity of TS. In addition, our results indicated that TS showed different strengths of influence on FasL, DR4, and pro-caspase-7 proteins in OVCAR-3 and A2780/CP70 cells. Caspase-3/7 activity assay showed that TS had a more significant influence on OVCAR-3 cells compared to A2780/CP70 cells. The varying strengths of TS’s inhibitory effects might results from the fact that A2780/CP70 cells have stronger chemoresistance than OVCAR-3 cells [46].

Angiogenesis is an essential process for the development of solid tumors that is regulated by endogenous pro-angiogenic and anti-angiogenic factors. Tumor cells secrete pro-angiogenic factors, such as VEGF and basic fibroblast growth factor. VEGF is the master regulator that controls the key step of the angiogenic process, particularly the endothelial cell growth, survival, permeability, and migration. Inhibition of the VEGF pathway for anti-angiogenic therapy in ovarian cancer is currently being investigated [16]. In previous studies, theasaponin E_1_ has been found to inhibit the expression of VEGF [35]. However, the impact of TS on angiogenesis in ovarian cancer has not yet been reported. To explore if TS hampered tumor induced angiogenesis, the VEGF protein content in the cell culture supernatant of TS treated OVCAR-3 and A2780/CP70 cells was tested. VEGF ELISA assay results indicated that TS strongly reduced secretion of VEGF in OVCAR-3 and A2780/CP70 cells. Treatment with 5 μg/mL TS caused a 79.29% and 71.43% decline of VEGF secretion in OVCAR-3 and A2780/CP70 cells, respectively. The expression of VEGF in ovarian cancer cells was higher than VEGF in benign or normal ovarian cells [16]. Western blotting analysis showed that TS decreased the expression of protein hVEGF. This data coincided with the results of VEGF ELISA assay. It is implied that TS might exert its anti-angiogenic function by down-regulating VEGF expression. HIF-1, a heterodimeric transcription factor, composed of HIF-1α and HIF-1β subunits, belongs to the basic-helix-loop-helix (bHLH), PAS (PER, ARNT, SIM) superfamily [18,21]. Previous studies have shown that HIF-1 can control the transcription of over 70 genes, including VEGF. This explains the important influence of HIF-1 on a broad range of cellular processes such as cell survival, apoptosis, vascularization, invasion, angiogenesis, metastasis, epithelial homeostasis, and drug resistance [18,56]. Recently, enhanced levels of HIF-1α protein have been detected in the cytoplasm and nuclei of 40% to 80% of human carcinoma cases [56]. Detailed immunohistochemistry research has indicated that dysregulation and overexpression of HIF-1α are heavily implicated in cancer biology, specifically in areas of vascularization and angiogenesis [2]. This study found that TS decreased the protein levels of HIF-1α, but had no influence on HIF-1β in both cell lines. HIF-1β is known as a common subunit of multiple bHLH-PAS proteins, which is rapidly degraded by proline hydroxylation, but HIF-1α will accumulate and heterodimerise with HIF-1β to form HIF-1 when cells are under hypoxic conditions [56]. The results are in accordance with the VEGF protein level and the secretion of VEGF content in the cell culture supernatant. It is speculated that TS down-regulated the transcription of VEGF through a pathway dependent upon HIF-1α. Part of the anti-proliferation activity of TS can be attributed to the anti-angiogenesis effect of TS that targets VEGF in an HIF-1α dependent manner.

## 4. Materials and Methods 

### 4.1. General Experimental Procedures

CellTiter 96^®^ Aqueous One Solution Cell Proliferation assay (MTS) and Caspase-Glo 3/7 Assay Kit were both obtained from Promega Corporation (Madison, WI, USA). Hoechst 33342 was purchased from Sigma-Aldrich (St. Louis, MO, USA). Dead Cell Apoptosis Kit with Annexin V Alexa Fluor^®^ 488 and propidium iodide (PI), and fetal bovine serum (FBS) were from Invitrogen (Grand Island, NY, USA). Quantikine Human VEGF Immunoassay Kit was purchased from R&D Systems (Minneapolis, MN, USA). Primary antibodies for Bad (C-7) (#8044), Bcl-xL (H-5) (#8392), and GAPDH (0411) (#47724) were from Santa Cruz Biotechnology, Inc. (Dallas, TX, USA). Bcl-2 (#2872S), HIF-1α (#14179S), hVEGF (#8065), FasL (#4273S), FADD (#2782S), DR5 (#3696S), monoclonal pro-caspase-9 (C9) (#9508S), pro-caspase-3 (#9662S), and pro-caspase-7 (#9492S) antibodies and horseradish peroxidase-conjugated secondary antibody were purchased from Cell Signaling Technology, Inc. (Danvers, MA, USA). Antibody HIF-1β (#611079) was from BD Biosciences (San Jose, CA, USA). The BCA Protein Assay Kit was obtained from Thermo Fisher Scientific (Waltham, MA, USA). Halt^TM^ Protease and Phosphatase Inhibitor Single-Use Cocktail were purchased from Life Technologies (Grand Island, NY, USA). EDTA solution was form Thermo Scientific (Rockford, IL, USA).

### 4.2. Cell Culture

The same cell lines were used as described in the previous studies in our team [37,42,43]. Briefly, human platinum-resistant ovarian cancer cell lines OVCAR-3 and A2780/CP70 were provided by Dr. Jiang from West Virginia University. IOSE-364, a normal ovarian surface epithelial cell line, was provided by Dr. Auersperg from the University of British Columbia, Vancouver, BC, Canada. All cells were cultured in RPMI-1640 medium supplemented with 10% FBS. Cells were cultured in a humidified incubator containing 5% CO_2_ at 37 °C. Then cells were subsequently treated with TS at various concentrations for a certain time adjusted by each experiment.

### 4.3. Extraction and Purification of TS 

**Figure 6:**
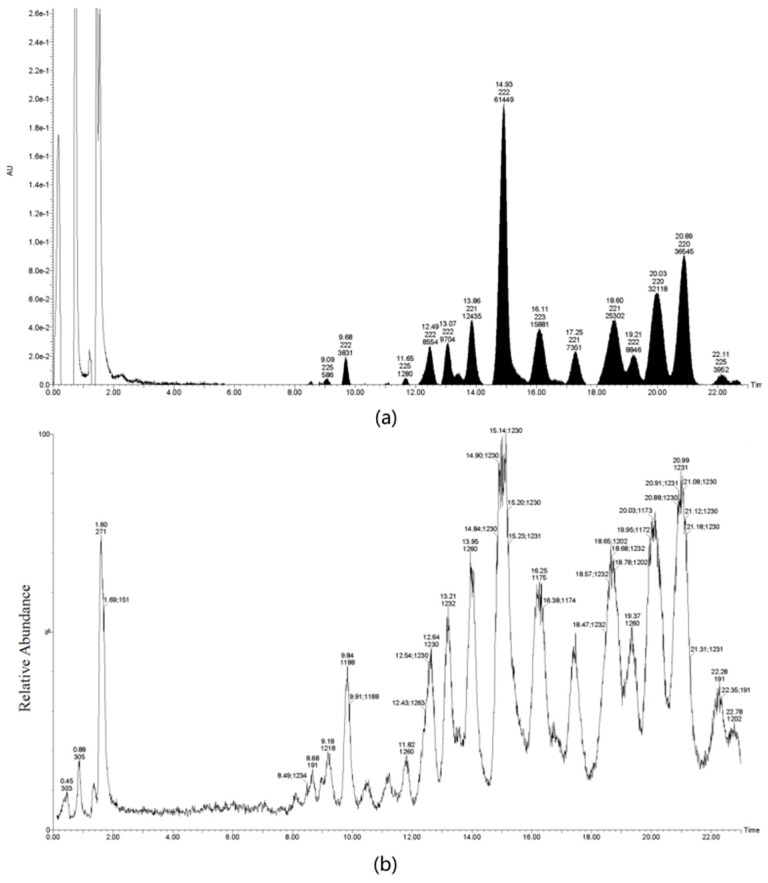
UPLC-MS analysis of TS isolated from green tea seeds. (**a**) Base peak intensity chromatogram at 210 nm; (**b**) Base peak intensity chromatogram at *m*/*z* 100–2000.

TS was isolated from Green tea (*Camellia sinensis* (L.) O. Kuntze) seeds, which were freshly harvested from the National germplasm Hangzhou tea tree nursery, Hangzhou, China, and modified by previously-established methods [36]. Briefly, the dried tea seeds were pulverized and subsequently extracted with 70% MeOH (1:15 *w*/*v*) under reflux for 5 h at 70 °C. The MeOH suspension was filtered, concentrated, and defatted twice with petroleum ether (1:1, *v*/*v*), then partitioned twice between H_2_O and EtOAc (1:1, *v*/*v*) to remove flavonoids, and then between H_2_O and BuOH (1:1, *v*/*v*) to yield dark-brown material. The crude extract was further passed through D101 macroporous resin and eluted with different concentrations of ethanol at 0%, 30%, 50%, 70%, and 90% to yield 40 fractions. Similar fractions were combined based on the HPLC profile. The most active fraction was further separated on a semi-preparative C_18_ column. We used 50% MeOH to remove persistent flavonoids and then 70% MeOH to yield TS. Then TS was characterized by UPLC-MS analysis (Figure 6) performed on a Acquity^TM^ Ultra performance LC instrument (Waters Corporation, Milford, MA, USA). We used Welch Ultimate^®^ UHPLC XB-C_18_ column (100 mm × 2.1 mm, 1.8 µm; Welch Materials, Inc., Shanghai, China). The UPLC system was carried out under the following conditions: Solvent A was acetonitrile and solvent B was distilled water containing 0.1% formic acid. The non-linear gradient system used was initially A/B (72:28) to A/B (56.8:43.2) at 7 min to A/B (56.5:43.5) for 4 min, then to A/B (52:48) for 5.5 min, held A/B (52:48) for 1.5 min, then to A/B (72:28) for 2 min, and held A/B (72:28) for 2 min; total time, 22 min; flow rate, 0.2 mL/min; and detection, UV 210 nm; column oven temperature, 25 °C. The MS system was carried out under following conditions: MS capillary voltage, 3 kV; sample cone, 90 V; source temperature, 150 °C; desolvation gas temperature, 500 °C; cone gas flow, 50 L/h; desolvation gas flow, 600 L/h; scan, *m*/*z* 100–2000.

### 4.4. Cell Viability Assay

The inhibition of cell growth was detected using MTS assay according to the manufacturer’s instructions based on the method as described before [37]. Briefly, all cells were seeded into 96-well plates at a density of 1.0 × 10^4^ cells/well in RPMI-1640 medium incorporation 10% FBS. Prior 16 h growth before cells were treated with different concentration of TS or cisplatin (0 to 20 μg/mL) for 24 h. Control cells received an equal amount of RPMI-1640 medium with FBS free. The aim of TS concentration used in MTS was to determine if the anti-proliferation of TS in a dose-dependent manner and set appropriate TS concentrations used in the subsequent assays. For MTS assay, 100 μL of MTS was subsequently added to each well and then incubated in the dark at 37 °C for at least 1 h. The absorbance was measured at 490 nm. Cell viability was shown as the percentage compared to control group. All data were collected from four independent experiments. Cytotoxicity of each sample was expressed as the concentration of test sample that causes 50% inhibition of cell growth (IC_50_).

### 4.5. Colony Formation Assay

Human ovarian cancer OVCAR-3 and A2780/CP70 cells (100 cells/well) were plated into 12-well plates and then cultured in RPMI medium containing 10% FBS at 37 °C, 5% CO_2_ in a humidified incubator 72 h before treated with TS (0, 1, 2.5, and 5 μg/mL) for 24 h. Afterwards, the supernatant was removed and then cells were cultured under RPMI medium with 10% FBS for another 10 days. Following that, the medium was wiped out and the cells were washed with PBS twice at room temperature. Crystal violet-stained cells for 15 min after formaldehyde fixing cells for at least 15 min. The numbers of colonies was counted under light microscope. The results were expressed as the mean ± standard deviation (SD) of three independent experiments.

### 4.6. Apoptosis Assessment by Hoechst 33342 Staining

Based on the method used in our team [42,43], briefly, OVCAR-3 and A2780/CP70 cells were seeded into 96-well plates at the density of 1.0 × 10^4^/well and incubated for 16 h prior. Then cells were treated with different concentrations (0 to 5 µg/mL) of TS or RPMI-1640 without 10% FBS (as the control) for 24 h. Subsequently, cells were stained with 10 µg/mL Hoechst 33342 in PBS for 10 min at 37 °C in the dark. Cell apoptosis was detected under a fluorescence microscope (ZEISS, Oberkochen, Germany), and data were collected from three to five replicate experiments.

### 4.7. Caspase-3/7 Assay

OVCAR-3 and A2780/CP70 cells were seeded into 96-well plates at the density of 2.0 × 10^4^ cells/well. After overnight growth, cells were treated in a medium lacking 10% FBS (as the control) and different concentrations (0 to 5 µg/mL) of TS for 24 h. After treatment, caspase-3/7 vitality in both cell lines was determined using a Caspase-Glo 3/7 Assay kit. The total protein levels were measured via a BCA Protein Assay Kit. Caspase-3/7 activities were normalized by total protein levels and were expressed as a percentage compared to the untreated group.

### 4.8. Flow Cytometry Analysis of Cell Apoptosis

According to the method described in the manufacturer’s instructions of Alexa Fluor 488 Annexin V/Dead Cell Apoptosis kit. In brief, both OVCAR-3 and A2780/CP70 cells (10^6^/dish) treated with TS or RPMI-1640 (FBS free) for 24 h were washed with cold PBS twice and digested by trypsin. Cells were collected by 1000 rpm centrifugation for 8 min at 4 °C. Then the cell pellets were re-suspended in 1 mL 1× Annexin V binding buffer. An aliquot of 100 µL of the cell solution (around 2.0 × 10^5^ cells) was transferred to a 1.5 mL tissue culture tube. Subsequently, 5 µL of Annexin V-FITC was added into tubes and gently vortexed and incubated in the dark at room temperature for 15 min. Then 10 µL PI (1 mg/mL in PBS) was added to each tube and stained for 10 min when kept on ice. Following this, 290 µL of binding buffer was added to each tube and gently vortexed before detection. The samples were analyzed by flow cytometry (FACSCalibur system, BD Biosciences, San Jose, CA, USA). Data was plotted and analyzed using FCS software (De Novo Software, Los Angeles, CA, USA).

### 4.9. Enzyme-Linked Immunosorbent Assay

Following the method decribed before [37], briefly, both human ovarian cancer cell lines OVCAR-3 and A2780/CP70 cells were seeded into 96-well plates at a density of 2.0 × 10^4^ per well, then incubated overnight and treated with TS at concentration range from 0 to 5 μg/mL for 24 h. After treatment, cell culture supernatants were collected for VEGF assay. VEGF protein levels were analyzed by sandwich enzyme-linked immuno-sorbent assay (ELISA) with a Quantikine Human VEGF Immunoassay Kit according to the manufacturer's instructions. Three independent experiments were assayed, and the mean VEGF protein level from each duplicate was used for statistical analysis.

### 4.10. Western Blotting

Human ovarian cancer cells (10^6^ cells/well) were seeded into 60 mm dishes and incubated for 16 h before treatment with different concentrations of TS (0,1, 2.5, and 5 μg/mL), RPMI-1640 without 10% FBS (as the control) for 24 h. After washing with PBS twice, cells were harvested and lysed with 100 μL of Mammalian Protein Extraction Reagent supplemented with 2 μL Halt^TM^ Protease, Phosphatase Inhibitor Single-Use Cocktail, and 2 μL EDTA solution. Then, all samples were frozen at −80 °C for at least 1 h before being centrifuged at 12,000× *g* at 4 °C for 15 min, and collected in the aqueous phase for measurement. A BCA Protein Assay Kit was used to determine the concentration of total protein levels. Equal amounts of protein were separated by 10% SDS-PAGE and transferred onto nitrocellulose (NC) membranes. Then the NC membranes were blocked with 5% non-fat milk in a Tris-buffer saline solution containing 0.1% Tween-20 for 1 h at room temperature and then incubated with the primary antibodies overnight at 4 °C. The next day, the NC membranes were incubated by the secondary antibody for 2 h at room temperature. Detection was performed by Super Signal West Dura Extended Duration Substrate (Life technologies, Grand Island, NY, USA) and Chemi Doc^TM^ MPSystem (Bio-Rad, Hercules, CA, USA). Protein bands were quantified with NIH ImageJ software (NIH, Bethesda, MD, USA) and normalized based on the GAPDH control for analysis.

## 5. Conclusions

To summarize, TS had potent inhibitory effects on cell proliferation and colony formation in vitro on both cisplatin-resistant ovarian cancer cell lines OVCAR-3 and A2780/CP70. The growth-inhibitory activities of TS were observed at concentrations as low as 1 μg/mL. However, TS had no significant cytotoxic effect on normal ovarian epithelial cells unless with a concentration higher than 20 μg/mL. TS displayed a strong selectivity in eliminating ovarian cancer cells and in sparing normal cells. The results showed that TS influenced both OVCAR-3 and A2780/CP70 cell-induced apoptosis and angiogenesis. The induction of apoptosis by TS was correlated with the extrinsic pathway through increased expression of DR5 and FADD and increased caspase-3/7 activation. The inhibited angiogenesis effect of TS was correlated with the down-regulation of HIF-1α and VEGF protein levels in ovarian cancer cells. The data suggests that TS may be regarded as an alternative anti-tumor agent for the adjunctive therapy of human ovarian cancer and could be a potential application for the therapy of human ovarian cancer in the future.
